# Rapid and Highly Sensitive Detection of Lead Ions in Drinking Water Based on a Strip Immunosensor

**DOI:** 10.3390/s130404214

**Published:** 2013-03-28

**Authors:** Hua Kuang, Changrui Xing, Changlong Hao, Liqiang Liu, Libing Wang, Chuanlai Xu

**Affiliations:** School of Food Science & Technology, State Key Laboratory of Food Science & Technology, Jiangnan University, Wuxi 214122, China; E-Mails: da_rui12345@163.com (C.X.); changlongup@163.com (C.H.); raxray@gmail.com (L.L.); wanglb1@126.com (L.W.); xcl@jiangnan.edu.cn (C.X.)

**Keywords:** amplified competitive lateral flow assay, lead ions, immunosensor, gold nanoparticles

## Abstract

In this study, we have first developed a rapid and sensitive strip immunosensor based on two heterogeneously-sized gold nanoparticles (Au NPs) probes for the detection of trace lead ions in drinking water. The sensitivity was 4-fold higher than that of the conventional LFA under the optimized conditions. The visual limit of detection (LOD) of the amplified method for qualitative detection lead ions was 2 ng/mL and the LOD for semi-quantitative detection could go down to 0.19 ng/mL using a scanning reader. The method suffered from no interference from other metal ions and could be used to detect trace lead ions in drinking water without sample enrichment. The recovery of the test samples ranged from 96% to 103%. As the detection method could be accomplished within 15 min, this method could be used as a potential tool for preliminary monitoring of lead contamination in drinking water.

## 1. Introduction

Lead contamination is a serious worldwide environmental problem. As it is difficult to detoxify by chemical or biological methods, gradual lead ion accumulation in the nervous and cardiovascular systems of the human body can subsequently cause serious diseases [1]. Young children are especially vulnerable by breathing or swallowing lead paint chips and dust. Over the past several decades, some areas in China have been suffering from serious heavy metals contamination at the cost of economy development and this has even directly influenced the supply of safe water for drinking and farming. In the U.S., the National Primary Drinking Water Regulations (NPDWRs) limit for the action level of lead ion contaminants in drinking water is 15 ng/mL. Long-term health consequences of drinking lead-contaminated water include kidney problems and high blood pressure for adults, and the physical and mental development delays in infants and children [2]. Lead contamination in water has attracted significant attention around the World, and fast and sensitive methods for monitoring water quality are for protecting human and animal health in great demand.

Although various new detection techniques have been developed through colorimetry, fluorimetry, and voltammetry [3–8], currently common and reliable detection methods for lead ions in water, vegetables and many other food products mainly depend on laboratory instrument analysis including graphite furnace atomic absorption spectrometry (GFAAS), inductively coupled plasma atomic emission spectroscopy (ICP-AES) [9] and inductively coupled plasma mass spectrometry (ICP-MS) [10]. These methods are expensive, time-consuming, labour-intensive and require professional staff to perform the testing, so a simple, economical and general detection method for direct analysis of drinking water samples is imperative.

ELISA has been developed as an effective alternative tool for the detection of some metal ions by many groups [11–14]. Zhou *et al.*[13] have demonstrated an improved method using gold nanoparticles as an amplified probe to detect Hg(II), Pb(II), and Cd(II) based on the traditional ELISA system. The detection process was still time consuming and involved multiple incubation and wash steps so this method could not satisfy the requirements for rapid and *on-site* detection. Recently, the lateral flow assay (LFA) has emerged as a powerful analysis platform for detecting various analytes including small molecules, such as pesticides, biotoxins and heavy metals, due to its speed, simplicity and low-cost characteristics [15–18]. However, a common problem is that the sensitivity of the conventional LFA is lower than that of the ELISA. Tang *et al.*[16] have used nanometer-sized TiO_2_ to enrich the lead ions in water samples before using a strip test. The sensitivity of this conventional LFA was low, which could not satisfy the detection requirements. While some enhanced methods have been applied to improve the sensitivity of the LFA, most of them were based on sandwich assays to detect large biological molecules [19], rather than on competitive assays to detect small molecules. In this report, we first introduce a heterogeneously-sized gold amplified probe to construct a strip immunosensor for detecting lead ions in drinking water samples. Compared with the conventional method, this amplified method has typically increased sensitivity by 4-fold with a visual limit of detection (LOD) of 2 ng/mL. This antibody-based sensor suffered from no interference from other metal ions. The detection process was simple and could be accomplished within 15 min without any enrichment process. This method could be used as a potential tool for preliminary monitoring of lead contamination in drinking water.

## 2. Materials and Methods

### 2.1. Chemicals and Equipment

Pb(II), Hg(II), Cd(II), Cu(II), Cr(III), Mn(II), Co(II), Fe(II), Zn(II), Al(III), Mg(II), and Ca(II) (1000 μg/mL in 1% HNO_3_ or 5% HCl) were all atomic absorption standards purchased from the National Institute of Metrology P.R China (Beijing, China). 1-(4-Isothiocyanobenzyl)ethylenediamine-N,N,N′,N& prime;-tetraacetic acid (ITCBE) was purchased from Dojindo Laboratories (Shanghai, China). Anti-Pb(II)-ITCBE monoclonal antibody and anti-OVA monoclonal antibody were produced in our laboratory [20,21]. Au NPs colloids were synthesized in our laboratory [22,23]. Keyhole limpet hemocyanin (KLH), bovine serum albumin (BSA), Freund's complete adjuvant, Freund's incomplete adjuvant and goat anti-mouse IgG antibody were obtained from Sigma Aldrich (Shanghai, China). The type of nitrocellulose (NC) membrane used was a Sartorius CN 140 and the glass fiber conjugate (GFC) pad was purchased from Whatman (Dassel, Germany). HEPES buffer solution (HBS) with 0.137 M NaCl, 3 mM KCl, and 10 mM HEPES, pH 7.4 was used in this study. Other chemicals were of high purity analytical grade and purchased from standard commercial reagent companies. All the solutions were prepared in ultrapure water obtaioned from a Milli-Q Ultrapure System.

A CM4000 Guillotine Cutting Module (BioDot Inc., Irvine, CA, USA) and Dispensing Platform (BioJet Quanti3000 dispenser) were used to manufacture the test strips. The BioDot TSR3000 Membrane Strip Reader was used to test the color intensities of colloidal gold on the test zone. The ELISA results were detected by a microplate reader (MK3, Thermo Labsystems, Chicago, IL, USA). ICP-MS (XSERIES 2, Thermo Fisher Scientific, Waltham, MA, USA) was used to detect lead ions in water samples.

### 2.2. Preparation of Protein-Chelate Conjugates

BSA (10 mg) or KLH (20 mg) was added in 0.1 M HEPES buffer solution (pH 9.0) containing 2 mM ITCBE, 2 mM lead ions. The final concentration of protein was adjusted to 2 mg/mL [24,25]. The reaction mixtures were stirred slightly overnight at room temperature and the pH was kept at 9.0. Then unreacted ITCBE and Pb(II)-ITCBE complexes were removed from the metal-chelate protein conjugates by ultrafiltration centrifugation at 8000 rpm for 30 min three times. The resulting Pb(II)-ITCBE-BSA and Pb(II)-ITCBE-KLH conjugates were stored in HBS (500 μL, 2 mg/mL) at −20 °C before use.

### 2.3. Immunization, Cell Fusion, and Purification of Monoclonal Antibodies

Five hundred μL of conjugated immunogen [Pb(II)-ITCBE-KLH] was emulsified with an equal volume of Freund's complete adjuvant. Nine six-week-old BALB/c mice were injected subcutaneously (100 μg per mouse). The injections were repeated three times at 3-week intervals with Freund's incomplete adjuvant and the injection dose was reduced in half. Ten days after the third injection, blood samples were collected from the tail of mouse for antibody titer and inhibition determinations by indirect competitive assay. The mouse showing highest serum reactivity with Pb(II)-ITCBE was selected to cell fusion according standard operating procedure [26]. Positive hybridoma clones synthesizing and secreting antibody to Pb(II)-ITCBE complex were screened and subcloned twice by limiting dilution. BALB/c mice primed with Freund's incomplete adjuvant were injected of 1.8 × 10^6^ hybridoma cells and ascites fluid was collected 12–14 days later. The ascites fluid was purified by the caprylic acid-ammonium sulfate method. Briefly, the purification is performed by adding 1 mL of ascetic fluid to 2 mL of 0.06 M acetate buffer, pH 4. After adjusting the pH of the solution to 4.8 with 1 M HC1, 33 μL of caprylic acid solution are added dropwise with vigorous stirring. Then the solution is stirred for 30 min at room temperature and centrifuged at 10,000 rpm for 30 min at 4 °C. The supernatant is harvested by centrifugation, adjusted to pH 5.7 with 1 M NaOH and dialyzed against 0.05 M acetate buffer, pH 5.7. Then the antibody was further purified by saturated ammonium sulfate and dialyzed against 0.01 M phosphate buffered saline, pH 7.4.

### 2.4. Competitive ELISA

Competitive ELISA [27] was performed to test different concentrations of lead ions in HBS buffer amended with 1 mM ITCBE for the ability to inhibit binding to the immobilized Pb(II)-ITCBE-BSA conjugate. The general procedure was as follows: microwell plates with 96 wells were coated (100 μL/well) with Pb(II)-ITCBE-BSA at 1.0 μg/mL in HBS buffer (HBS, 10 mM sodium HEPES, 3 mM KCl, 137 mM NaCl, pH 9.0) for 2 h at 37 °C. After being washed three times with phosphate buffered saline (PBS, 137 mM NaCl, 3 mM KCl, 10 mM phosphate, pH 7.4) containing 0.05% Tween 20 (PBST), the plates were blocked with 2% BSA in HBS for 2 h at 37 °C. After being washed again, the plates were air-dried 15 min at 37 °C. Fifty μL of purified antibody (1:30,000) was incubated in the presence of 50 μL of different concentrations of lead ions in HBS buffer amended with 1 mM ITCBE. After 0.5 h at 37 °C, the plates were washed with PBST and the amount of antibody captured by the Pb(II)-ITCBE-BSA was bound by the goat anti-mouse IgG-horseradish peroxidase (HRP) conjugate. After washing to separate the unbound goat anti-mouse IgG-HRP conjugate, 3,3′,5,5′-tetramethylbenzidine (TMB) substrate was added and oxidised by HRP into the final product. The results were read by the microplate reader at 450 nm.

### 2.5. Preparation of the Au NPs Conjugates

To prepare the detection probe, anti-Pb(II)-ITCBE monoclonal antibody (6 μL of 1 mg/mL) and anti-OVA monoclonal antibody (10 μL of 1 mg/mL) were diluted in borate buffer (0.1 M, pH 8.5) and added to 1 mL of colloidal gold solution (∼10 nm diameter, TEM as seen in Figure S1, Supporting Information) with K_2_CO_3_ (10 μL of 0.5 M). After vibration for 30 min at room temperature, 0.05 mL of 100 mg/mL BSA in PBS was added to the solution to block the Au NPs surface. Following incubation for 2 h at room temperature, the mixture was centrifuged at 11,500 rpm for 10 min. The supernatant was discarded and the Au NPs conjugate was suspended in PBS containing 2% (w/v) BSA, 2% (w/v) sucrose and 0.02% (w/v) sodium azide. The centrifugation and suspension processes were repeated, and the final volume of 200 μL was stored at 4 °C until use. The preparation process of the amplified probe, goat-anti mouse IgG labeled Au NPs, was the same as that of detection probe. The differences were that the diameter of Au NPs used was 30 nm (Figure S1, Supporting Information) and corresponding centrifugation speed was 7500 rpm.

### 2.6. Competitive Lateral Flow Assay

**Figure 1.**
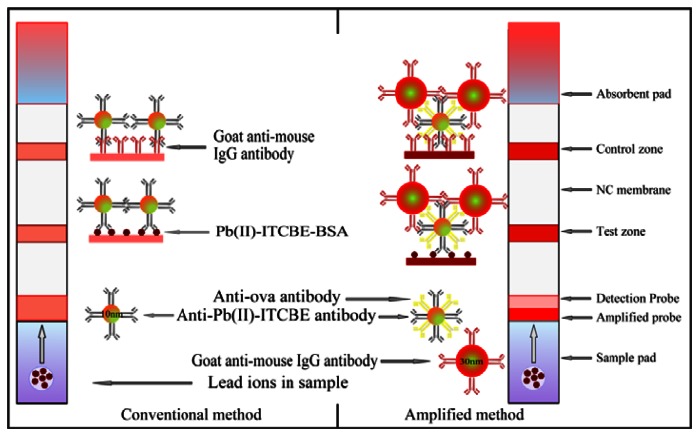
Schematic illustration of the principle of visual detection for lead ions with a conventional and an amplified lateral flow strip immunosensor.

The LFA strip for the amplified detection of lead ions was constructed from a NC membrane, a sample pad, an absorbent pad, and two conjugate pads as shown in Figure 1. The width of the plate was cut to 4 mm. The NC membrane was pasted on the plastic backing support board. The goat anti-mouse IgG (0.5 mg/mL) and Pb(II)-ITCBE-BSA (1 mg/mL) were added to the NC membrane as the control line and test line by the dispensing platform (BioJet Quanti3000 dispenser) and air-dried for 2 h at 37 °C. The sample pad was saturated with a solution containing 0.05% (w/v) Tween, 2% (w/v) sucrose and 0.1% (w/v) sodium azide and then air-dried 4 h at 37 °C. The detection probe (10 μL/strip) and the amplified probe (10 μL/strip) were added to the glass fiber membrane respectively and air-dried 1 h at 37 °C. The amplified probe was placed on the top of the detection probe. The integrated strips were placed in the plastic housing and stored in the hermetic bag with desiccating agent. A conventional LFA strip was also prepared in the same manner without the amplified probe.

### 2.7. Sensitivity and Cross-Reactivity Testing

The strip sensor was applied to the detection of different concentrations of lead ions. Briefly, 50 μL of lead standard solution (0, 0.25, 0.5, 1, 2, 4 and 8 ng/mL) was added to 50 μL 2 mM ITCBE in 2× HBS buffer solution (20 mM sodium Hepes, 6 mM KCl, 274 mM NaCl, pH 7.4) and incubated at 37 °C for 0.5 h after a few minutes stir. Then 100 μL of mixture solution was pipetted into the sample port of the strip. The standard solution with different concentrations of lead ions was tested by the conventional and amplified method separately. The results were determined on the basis of presence/absence of the test line after 10 min. The color intensity of the test zone on the strip paper was recorded by the BioDot TSR3000 Membrane Strip reader at the same time (Gene Company Limited, Shanghai Branch, Shanghai, China). To evaluate the specificity of this method, metal ions including Hg(II), Cd(II), Cu(II), Cr(III), Mn(II), Co(II), Fe(II), Zn(II), Al(III), Mg(II), and Ca(II) were tested for cross-reactivity at the concentration of 1000 ng/mL.

### 2.8. Sample Analysis

Specific concentrations of lead ions were spiked in drinking water samples to apply this method to test environment samples. All drinking water samples were filtered using a 0.45 nylon filter and the pH was adjusted to 7.4. The treatment of drinking water sample was the same as described before. The spiked samples and nonspiked samples were all detected by the strip three times.

## 3. Results and Discussion

### 3.1. Characterization of Anti-Pb(II)-ITCBE Monoclonal Antibody

The indirect competitive assay was performed and the lead ions concentration that produces a 50% inhibition in the signal (IC_50_) was 1.3 ng/mL. (see Figure S2, Supporting Information). The antibody specificity was tested and no cross-reactivity was found for Hg(II), Cd(II), Cu(II), Cr(III), Mn(II), Co(II), Fe(II), Zn(II), Al(III), Mg(II), and Ca(II), even at a concentration of 1000 ng/mL (data not shown). The antibody demonstrated sensitivity and specificity suitable for the lateral flow assay.

### 3.2. Principle and Optimization

The scheme of the amplified method was showed in Figure 1. When an analyte solution is first applied to the sample pad and passes the amplified probe and the detection probe successively, the conjugates can be rehydrated and spread on the NC membrane by capillary action. The detection probe will move faster than the amplified probe as different sizes of the Au NPs used. The detection probe captured by the Pb(II)-ITCBE-BSA will interact with the amplified probe to make more Au NPs aggregate on the test zone. The accumulations of Au NPs will form obvious red bands on the test zones which could be used for qualitative determination. The color intensity will decrease as the concentration of the analyte increases. The different intensities of the bands were recorded with the strip reader and the peak areas from the test zones were used for quantitative analysis. To measure the sensitivity of the method, the visual detection limit of the strip was defined as the minimum target concentration making the color on the test line disappearing. The LOD by the scanning reader of the strip for quantitative detection was calculated by the standard curve plotted as a function of lead ions concentration.

**Figure 2.**
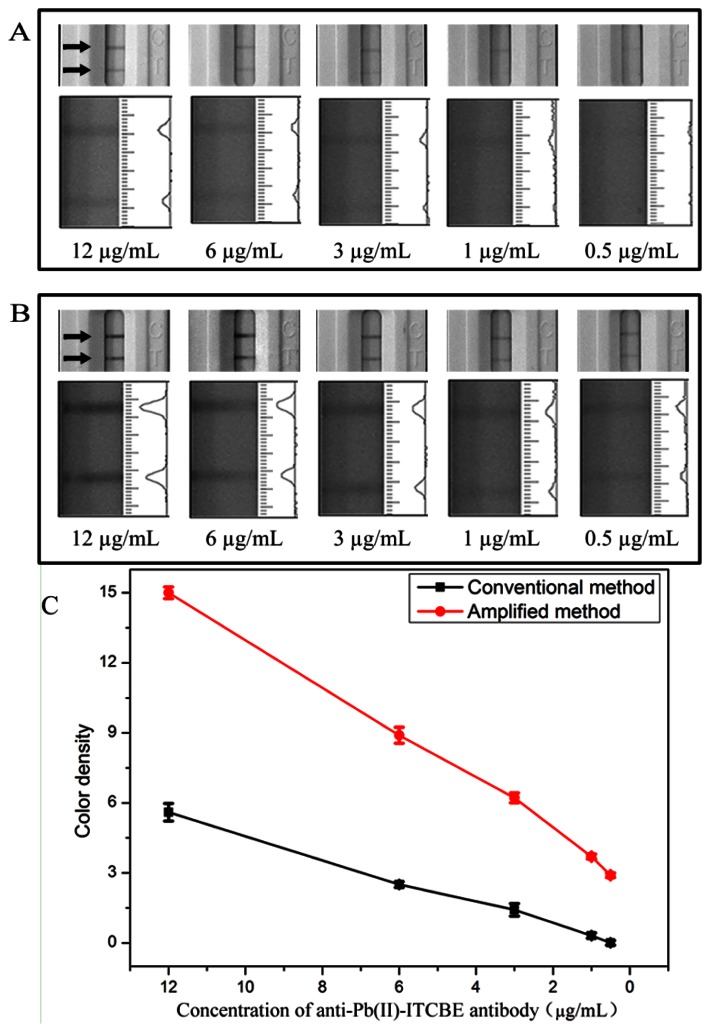
Effect of the concentration of antibody (12, 6, 3, 1 and 0.5 μg/mL) on the color development of the LFA. Detection results of the conventional method (**A**) and the amplified method; (**B**) with no analyte; (**C**) the resulting calibration curves. The corresponding optical responses of red bands on the strip were recorded with a strip reader. Each sample was analyzed for three replicates and error bars represent the standard deviations.

Typically, the LFA format used for small molecule detection is an indirect competitive method through ‘turn-off’ approaches. The intensities of the bands will decrease as the concentration of lead ions increase. However, the amplified probe will deepen the intensities of the bands on the test zone through ‘turn-on’ approaches making the sensitivity decrease. In order to increase sensitivity of the competitive LFA, the detection probe used in the amplified method was optimized. As shown in Figure 2, the concentration of anti-Pb(II)-ITCBE antibody was optimized from 12 μg/mL to 0.5 μg/mL (a typical photo is seen in Figure S3, Supporting Information). As can be seen, the intensities of the bands on the test zone decreased when lowering the concentration of antibody (Figure 2(A)). The results indicated that the amount of antibody conjugated onto the Au NPs surface dwindled. In order to capture more amplified probe and deepen the color intensity, anti-OVA monoclonal antibody was used to occupy the remaining position on the surface of Au NPs. As shown in Figure 2(B), the color intensities of the bands in the amplified method increased distinctly compared with that in the conventional method (Figure 2(A)).

**Figure 3.**
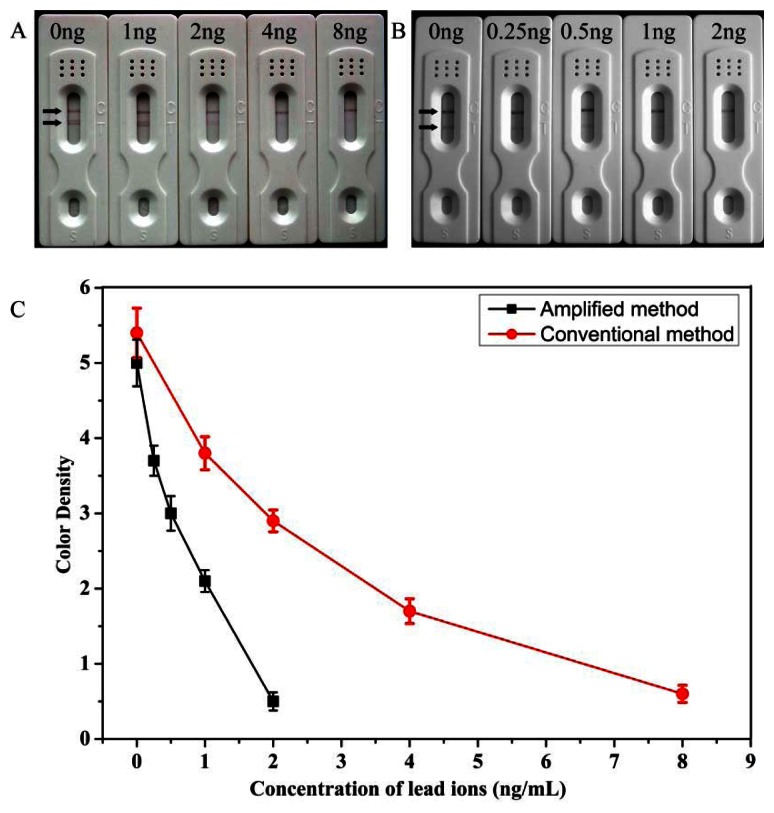
Detection results of the conventional LFA (**A**) and the amplified LFA; (**B**) with varying concentrations of lead ions; (**C**) the resulting calibration curves. The optical responses of red bands on the test zones of the strip were recorded with a strip reader. Each sample was analyzed for three replicates and error bars represent the standard deviations.

When 0.5 μg/mL of anti-Pb(II)-ITCBE antibody was used, no color was observed on the test zone in the conventional method and through amplification the color intensity on the test zone appeared. However, the line under these conditions was too faint for use as a negative control. In order to make the color intensities remarkable and comparable with that of the conventional method, an anti-Pb(II)-ITCBE antibody concentration of 3 μg/mL was used. The anti-OVA antibody of 10 μg/mL was used and this concentration was in excess. As shown in Figure 3, under these conditions, the color intensity of the negative results of the two methods were almost the same and the sensitivity was improved.

### 3.3. Sensitivity and Stability of the Detection

Under the optimized detection conditions, standard lead ions solutions of different concentrations were analyzed by the conventional and amplified LFA. Each sample was detected three times. A scanning reader was used to measure the intensity of the signal on the test zone and the corresponding optical responses curves are shown in Figure 3(C). As shown in Figure 3(A), we first analyzed different concentrations of lead ions (0, 1, 2, 4, 8 ng/mL) using the conventional LFA. The LOD was determined to be 8 ng/mL by visual inspection and the LOD for semi-quantitative detection could be as low as 0.81 ng/mL using the scanning reader. In the optimized amplification assay, different concentrations of lead ions (0, 0.25, 0.5, 1, 2 ng/mL) was tested (Figure 3(B,C)). The amplified method showed a good correlation of R^2^= 0.96, the linear range of detection was 0.25–2 ng/mL. The visual LOD was determined to be 2 ng/mL and the LOD for semi-quantitative detection could be as low as 0.19 ng/mL using the scanning reader. These results could meet the NPDWRs requirements for monitoring drinking water. Moreover, the sensitivity was increased 4-fold through the process of amplification. Compared with the previously reported methods for detecting of lead ions using the LFA reported by another group [16], our method has the lowest LOD in detecting lead ions without enrichment and was suitable for the *on-site* detection of trace concentrations of lead ions in drinking water.

Furthermore, the repeatability and reproducibility of the strip prepared in the same batch were evaluated. Strips sealed in a hermetic bag with desiccating agent were stored for 10 days, 20 days, and 30 days at room temperature, respectively. The drinking water samples without lead ions were tested using these strips. There are no significant differences observed between the test zones of the 10, 20 and 30 days strips, which showed that the strips were still viable following at least 30 days of storage.

### 3.4. Specificity Confirmation

**Figure 4.**
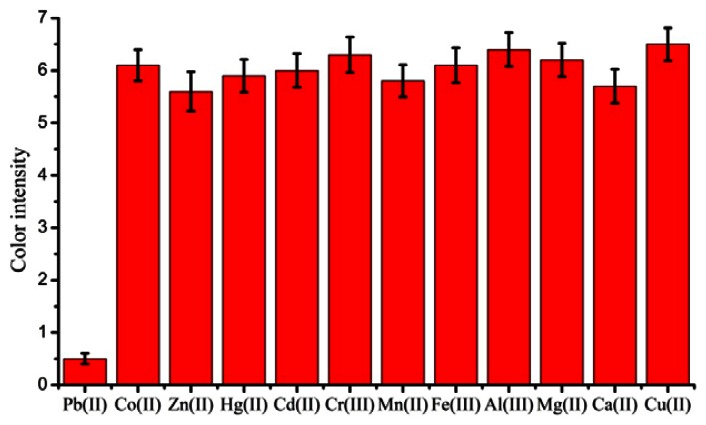
Cross-reactivity of other metals (1000 ng/mL) tested by strip immunoassay. The optical responses of red bands on the strip were recorded with a strip reader. Each sample was analyzed for three replicates and error bars represent the standard deviations.

To examine the cross-reactivity of the LFA, other metal ions Hg(II), Cd(II), Cu(II), Cr(III), Mn(II), Co(II), Fe(II), Zn(II), Al(III), Mg(II), and Ca(II) were tested. The results are shown in Figure 4. The test line did not disappear when the concentrations of these metal ions was up to 1000 ng/mL. This showed that the assay was highly specific and could be used for the rapid detection of low concentrations of lead ions.

### 3.5. Detection of Lead Ions in Drinking Water Samples

To evaluate the practicability of the strip sensor, we used drinking water as diluent. The drinking water sample was taken from bottled water purchased at the local market. Different concentrations of lead ions spiked in the drinking water samples were tested by the amplified LFA. The results are summarized in Table S1. The recoveries ranged from 96% to 103%, which would meet the detection requirements. Therefore, the strip could be used as a preliminary screening method for lead ions detection in water samples.

## 4. Conclusions

In this study, we have developed a rapid and sensitive strip immunosensor for the detection of lead ions in water samples. The visual LOD for qualitative detection of the amplified method was 2 ng/mL and the LOD for semi-quantitative detection could be as low as 0.19 ng/mL using a scanning reader. The recovery of the test samples ranged from 96% to 103%. This method could be used as a potential tool for preliminary monitoring of lead contamination in drinking water as the detection process was simple and could be accomplished in 15 min.

## Supplementary Material
