# Supplementary material for: Rapid and Highly Sensitive Detection of Lead Ions in Drinking Water Based on a Strip Immunosensor
Source: Sensors (Basel). 2013 Mar 28;13(4):4214–24. doi: 10.3390/s130404214 (PMC3673080; doi:10.3390/s130404214)
Supplement: Supplementary file 1 [file sensors-13-04214-s001.pdf]

OPEN ACCESS
sensors
ISSN 1424-8220
www.mdpi.com/journal/sensors

Supplementary Information

Rapid and Highly Sensitive Detection of Lead Ions in Drinking Water Based on a Strip Immunosensor. *Sensors* 2013, *13*, 4214-4224

Hua Kuang *, Changrui Xing, Changlong Hao, Liqiang Liu, Libing Wang and Chuanlai Xu

School of Food Science & Technology, State Key Laboratory of Food Science & Technology, Jiangnan University, Wuxi 214122, China; E-Mails: da_rui12345@163.com (C.X.); changlongup@163.com (C.H.); raxray@gmail.com (L.L.); wanglb1@126.com (L.W.); xcl@jiangnan.edu.cn (C.X.)

* Author to whom correspondence should be addressed; E-Mail: khecho@163.com; Tel.: +86-510-8532-9076.

Figure S1. TEM pictures of typical gold nanoparticles of 10 nm (**top**) and 30 nm (**bottom**) in diameter.

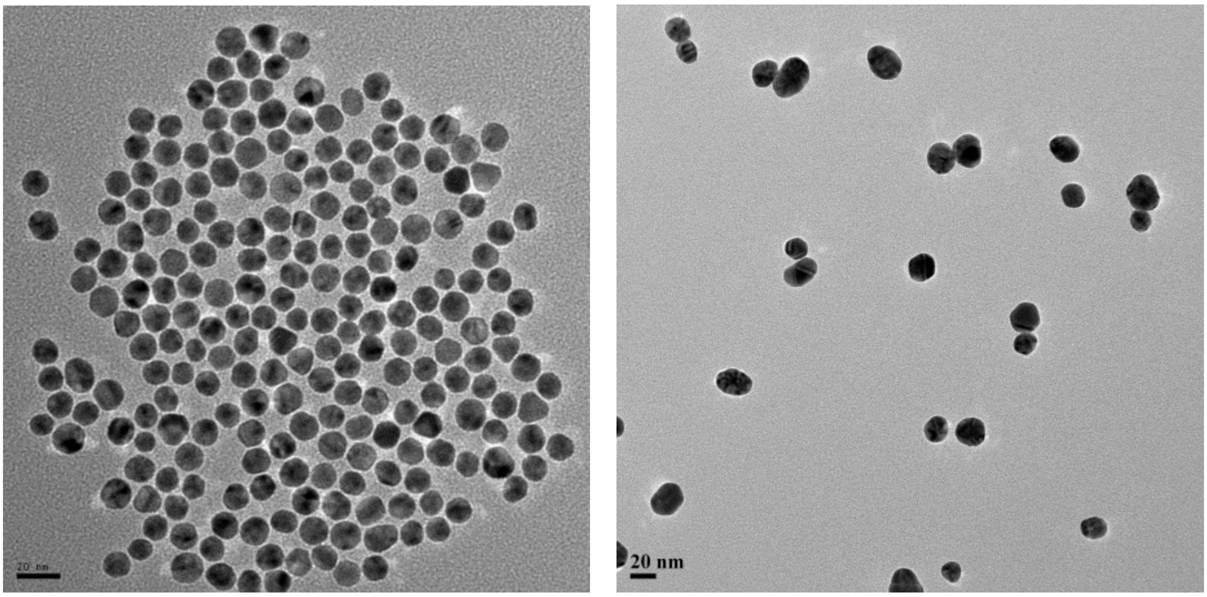

Figure S2. Standard inhibition curve of indirect competitive ELISA.

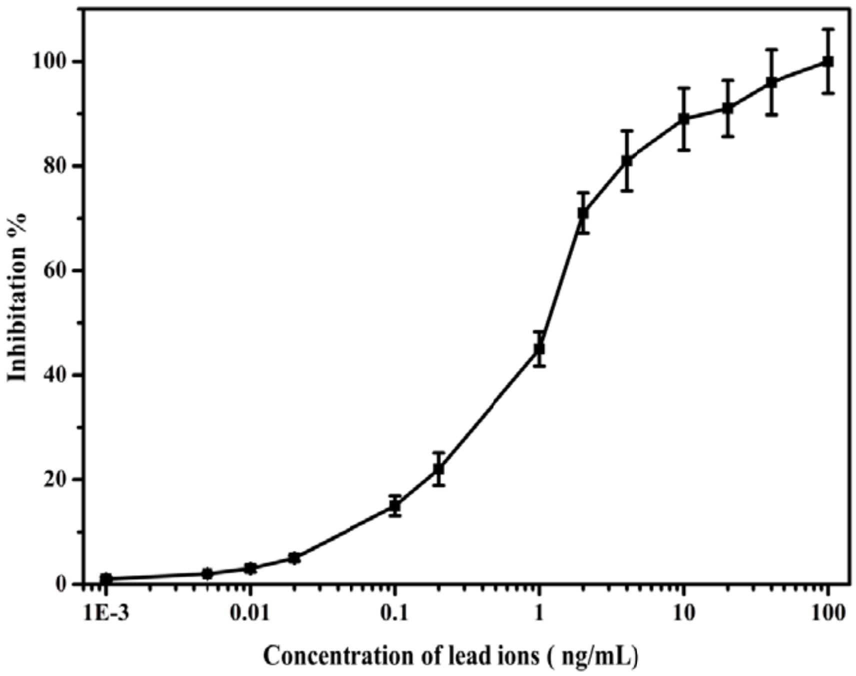

Figure S3. Typical photo images of detection results of the conventional method (**top**) and the amplified method (**bottom**) with no analyte.

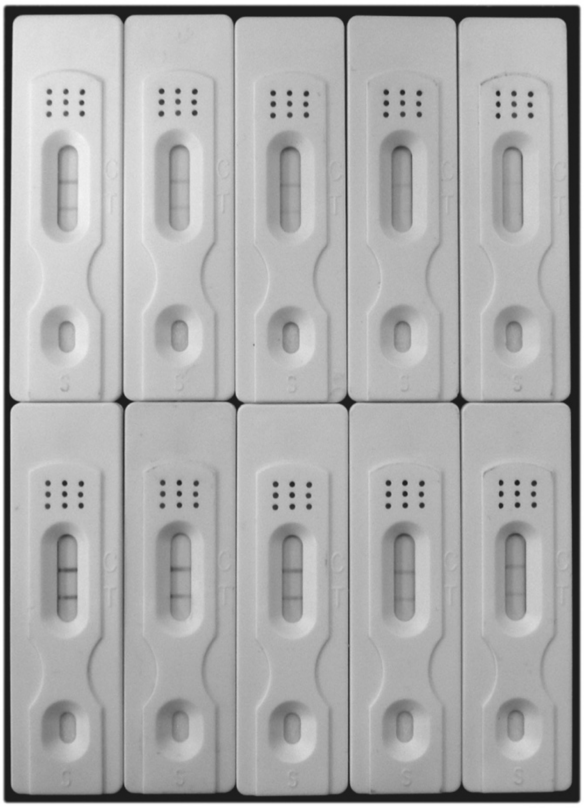

Table S1. Recovery test of lead ions in drinking water samples.

**Drinking Water Samples**	**Original Concentration [a] (ng/mL)**	**Spiked Concentration (ng/mL)**	**Detected Concentrations (Mean ± SD, n = 3) (ng/mL)**	**Recovery (%) (Mean ± SD, n = 3)**
1	1.2	0.25	1.44 ± 0.020	97.3±8.3
2	1.2	0.5	1.68 ± 0.043	96±8.7
3	1.2	1	2.17 ± 0.088	97±8.8
4	1.2	2	3.27 ± 0.19	103±9.6

[a] Original concentrations were detected by ICP-MS.

© 2013 by the authors; licensee MDPI, Basel, Switzerland. This article is an open access article distributed under the terms and conditions of the Creative Commons Attribution license (http://creativecommons.org/licenses/by/3.0/).
